# ADAMDEC1 accelerates GBM progression *via* activation of the MMP2-related pathway

**DOI:** 10.3389/fonc.2022.945025

**Published:** 2022-09-12

**Authors:** Huimin Qi, Ping Wang, Hongliang Sun, Xiaohan Li, Xinwei Hao, Wenxiu Tian, Liting Yu, Jiajian Tang, Junhong Dong, Hongmei Wang

**Affiliations:** ^1^ School of Basic Medicine, Weifang Medical University, Weifang, China; ^2^ Center of Translational Medicine, Zibo Central Hospital, Zibo, China; ^3^ School of Pharmacy, Binzhou Medical University, Yantai, China; ^4^ School of Medicine, Southeast University, Nanjing, China

**Keywords:** gliomas, glioblastoma multiforme, lower-grade glioma, a disintegrin and metalloproteinase domain-like protein decysin-1, matrix metalloproteinase 2

## Abstract

The ADAM (a disintegrin and metalloprotease) gene-related family including ADAM, ADAMTS, and ADAM-like decysin-1 has been reported to play an important role in the pathogenesis of multiple diseases, including cancers (lung cancer, gliomas, colorectal cancer, and gastrointestinal cancer). However, its biological role in gliomas remains largely unknown. Here, we aimed to investigate the biological functions and potential mechanism of ADAMDEC1 in gliomas. The mRNA and protein expression levels of ADAMDEC1 were upregulated in glioma tissues and cell lines. ADAMDEC1 showed a phenomenon of “abundance and disappear” expression in gliomas and normal tissues in that the higher the expression of ADAMDEC1 presented, the higher the malignancy of gliomas and the worse the prognosis. High expression of ADAMDEC1 was associated with immune response. Knockdown of ADAMDEC1 could decrease the proliferation and colony-forming ability of LN229 cells, whereas ADAMDEC1 overexpression has opposite effects in LN229 cells *in vitro*. Furthermore, we identified that ADAMDEC1 accelerates GBM progression *via* the activation of the MMP2 pathway. In the present study, we found that the expression levels of ADAMDEC1 were significantly elevated compared with other ADAMs by analyzing the expression levels of ADAM family proteins in gliomas. This suggests that ADAMDEC1 has potential as a glioma clinical marker and immunotherapy target.

## Introduction

Gliomas are one of the most lethal malignant tumors all over the world. There are different types of gliomas, including astrocytomas, brain stem gliomas, ependymomas, mixed gliomas (also called oligo-astrocytomas), oligodendrogliomas, and optic pathway gliomas. High-grade astrocytomas, called glioblastoma multiforme, are the most malignant of all brain tumors. The prognosis may vary depending on the type and location of the gliomas. Patients with a glioblastoma may have a survival rate of less than 1 year. The prognosis of patients with high-grade gliomas remains very poor because of cellular heterogeneity, therapeutic resistance, and a highly infiltrative nature. Two major types of glioblastomas based on mutations in the isocitrate dehydrogenase (IDH) 1 or 2 genes, and IDH-wild-type glioblastomas account for more than 90% of the cases according to WHO classification (1). Due to the invasive growth of malignant gliomas and the fragility of the central nervous system, surgery cannot completely remove the entire tumor from the brain, which brings great difficulties to the treatment of gliomas (2). Therefore, it is urgent to find a novel strategy for the treatment of glioma.

The disintegrin and metalloproteinase (ADAM) family is a class of glycoproteins anchored to the cell membrane that contain metalloprotease and disintegrin functional domains. There are more than 30 ADAM members involved in fertilization, neurogenesis, muscle development, immune response, and so on (3). In mechanism research, it also plays an important role in the hydrolysis of extracellular matrix, cell–cell and matrix adhesion, cell fusion, membrane protein shedding, proteolysis, and intracellular/extracellular signal transduction (4). Inflammation is a basic immune response including the basis of various physiological and pathological processes that prevent bacterial infection and repair damaged tissues (5). Cell–cell and cell–matrix interactions are critical in inflammation-related diseases. The dysregulation of ADAMs is associated with autoimmune diseases, inflammation, and cancers (6). Studies have found that inflammation is usually accompanied by the shedding of many transmembrane proteins. ADAMs can rapidly regulate key cell signaling pathways to induce the shedding of surface-associated factors to adapt to changes in the external environment. Furthermore, ADAMs disrupt cell adhesion and accelerate cell necrosis, and the shed protein fragments trigger inflammation (7–10). Cai et al. found that ADAMs are activated in the early stage of cell necrosis. Cell death is inhibited by knocking down ADAM9/10, which means that the activation of ADAMs may damage the cell epithelial barrier and exacerbate inflammation *in vivo* (8). Jones also found that ADAMs regulate intestinal development by controlling intestinal inflammation by shedding tumor necrosis factor receptor (TNFR) (11). Many studies have shown that ADAM17 is a key factor regulating the body’s injury and inflammatory response, and it is also a key factor leading to the reduction of cell adhesion (12–15). It may become a new target for cell necroptosis.

ADAMDEC1 (a disintegrin and metalloproteinase domain-like protein decysin-1) is also a member of the ADAM family, which is located on chromosome 8p12. ADAMDEC1 is the only metalloprotease in human and other mammalian genes that has aspartate residues instead of non-histidine zinc fingers (3). The ADAM family is involved in the maintenance of the malignant phenotype of glioblastoma and is expected to provide new ideas for the treatment of gliomas (16). Several studies reported that ADAM9, ADAM10, and ADAM17 showed high expression in glioma; meanwhile, ADAM9, as a potential regulator of glioma invasion, showed higher expression in GBM compared with LGG (17–19). ADAMDEC1, like other ADAM family members, also played an important role in the pathology of many diseases, most likely through regulating inflammation. Skin samples from patients with rosacea have a high expression level of ADAMDEC1. Furthermore, in the mouse model induced by antimicrobial peptide LL-37, silencing ADAMDEC1 reduced the expressions of IL-6, iNOS, and TNF-α, therefore reducing inflammation level (20). In the dextran sodium sulfate (DSS)-induced colitis mouse model, both the mRNA and protein levels of ADAMDEC1 were significantly higher in colonic mucosal platelet-derived growth factor receptor-alpha (PDGFRα)-positive cells compared with other cell types in the colonic mucosa, which suggested that this gene might be a potential drug target for colitis (21). Moreover, ADAMDEC1 has also been reported to contribute to the development of cancer. *In vitro*, ADAMDEC1 was shown to be a potential protein regulating gastric cancer cell proliferation and migration (22). Hwang et al. also found that the expression of ADAMDEC1 protein decreased when the migration of SK-Hep1 cells was inhibited (23). Macartney-Coxson et al. found that the destruction of colorectal cancer progressed rapidly with high ADAMDEC1 expression, and ADAMDEC1 can increase the metastatic potential of colorectal cancer (24). Supiot et al. confirmed that ADAMDEC1 protein is closely related to protein metabolism and cell adhesion in rectal cancer (25). For central nervous system tumors, it was found that ADAMDEC1 expression level was significantly higher than the control group in either enamel epithelial craniopharyngioma or squamous papillary craniopharyngioma. ADAMDEC1 is rarely expressed in grade I and grade II gliomas, but highly expressed in grade III and grade IV gliomas (26). Based on the important roles that ADAMDEC1 played in other cancers, this study investigated the contribution and prognostic signature of ADAMDEC1 in glioma. Our data suggested that ADAMDEC1 could serve as a glioma clinical marker and immunotherapy target.

## Materials and methods

### Software

UALCAN (http://ualcan.path.uab.edu/index.html), Kaplan–Meier Plotter database (https://kmplot.com/analysis/), EMBL-EBI (https://elixir-europe.org/about-us/who-we-are/nodes/embl-ebi), and TIMER (https://cistrome.shinyapps.io/timer/) were used to comprehensively analyze genomic features, overall survival, tumor immunity, and clinical aspects, and so on (27–30).

### Cell culture

The human glioma LN229, U87, U251, and T98G cell lines were obtained from the American Type Culture Collection. We had identified the LN229 cells by STR; they meet the requirements of the experiment. Cells were cultured in Dulbecco’s modified Eagle medium with high glucose (Life Technologies, Carlsbad, CA, USA) containing 10% fetal bovine serum (FBS, Gibco BRL). The medium was supplemented with penicillin (100 U/ml) and streptomycin (100 mg/ml). All cells were cultured at 37°C in 5% CO_2_ (31).

### Construction of short hairpin RNA targeting ADAMDEC1

The construction of the interference sequence targeting ADAMDEC1 gene was completed by Shanghai Genechem Co. Ltd. Three interfering sequences were designed according to the cDNA sequence of GenBank ADAMDEC1 (NM_014479), and then the most significant interfering sequence was screened by pre-experiment, shRNA-ADAMDEC1 5’-TACCACGAAACCTGAGAACAT-3’. Negative control sequence: 5’-TTTCCGAACGTGTCACGT-3’. Two complementary DNA single strands were synthesized and annealed to form double strands, ligated to the linearized vector GV493 (32), and the virus was packaged in HEK293T cells. When the confluency of glioma LN229 cells reached 30%–40%, they were infected with negative control lentivirus (shRNA-Con group) and shRNA-ADAMDEC1 lentivirus (shRNA-ADAMDEC1 group), and screened for stable transfection by the puromycin (4 μg/ml)-stained cell line.

### Construction of targeted ADAMDEC1 overexpression

The full-length ADAMDEC1 cDNA was obtained by PCR using the following primers: 5’ATGCTGCGTGGGATCTCCCAGC3’ and 5’TCACTCTGTGGTATGGTTTGGAGC3’, *Kpn*I/*Xho*I restriction sites were added at both ends, and *Kpn*I/*Xho*I was digested and then ligated into the pcDNA3.1 vector (Invitrogen, San Diego, CA). After sequencing confirmation, the empty vector pcDNA3.1 (Vector group) and the overexpression ADAMDEC1 plasmid (overexpression group) were transfected into LN229 cells by Lipofectamine 2000 (Invitrogen, Carlsbad, CA, USA). Stably transfected cell lines were screened by G418.

### RNA extraction and qRT-PCR

The total RNA of each group of glioma cells was extracted by TRIzol reagent (Qiagen, USA), and then the RNA purity and concentration of the cells were determined. RNA was reverse transcribed into cDNA using PrimeScript™ RT Master Mix (Takara Biotechnology Co., Ltd.). Real-time quantitative PCR was performed using the SYBR^®^ Premix Ex Taq™ II kit (Takara Biotechnology Co., Ltd.) following the manufacturer’s instructions. The primer sequences were as follows: ADAMDEC1: 5’-CAGTGTGTGGGAACCACCTT-3’ (forward) and 5’-GAGCATCTCCTCCGCAATCA-3’ (reverse); GAPDH: 5’-GCACCGTCAAGGCTGAGAAC-3’ (forward) and 5’-TGGTGAAGCGCCAGTGGA-3’ (reverse). The experiment was repeated three times, GAPDH was used as the internal reference, and the relative expression level of AMDEC1 gene was calculated by the 2^−ΔΔCt^ method.

### CCK-8 proliferation assay

LN229 cells were seeded at 3×10^3^/well in a 96-well plate and cultured in a 37°C, 5% CO_2_-saturated humidity incubator. Cell proliferation was then detected at 1, 2, 3, 4, and 5 days, respectively. Finally, 10 μl of the CCK-8 reagent (biosharp, BS350A) was added into each well according to the instructions of the kit, and to incubate at 37°C for 2 h. An automatic microplate reader was used to detect the absorbance of each well at 450 nm (D) value.

### Colony formation assay

Cells in each group were evenly seeded in six-well plates (500 cells/well) and routinely cultured at 37°C and 5% CO_2_ for 10 days. The cells were fixed with 4% paraformaldehyde for 30 min at room temperature and stained with 0.1% crystal violet for 20 min. The fixed cells were washed with 1× PBS and dried at room temperature. Next, we photographed and recorded the fixed cells. The number of clone colonies was counted using ImageJ software.

### Cell wound healing assay

The treated LN229 cells were seeded into a six-well plate (2.5×10^5^/ml). After 24 h, a sterile 10-μl pipette tip was used to make a smooth scratch on the monolayer of cells perpendicular to the well plate. The detached cell debris was washed with 1× PBS and photographed under an inverted microscope at 0 h and 24 h. The scratch healing rate was shown according to the ratio of the area of cell scratch healing to the initial scratch area within 24 h.

### Cell invasion assay

Matrigel working dilutions were prepared in a 1:5 ratio and coated with Transwell chambers. The treated cells were resuspended in serum-free medium, 2.5×10^5^ cells/200 μl were seeded into the upper chamber of Transwell™, and 600 μl of complete medium containing 10% serum was added to the lower chamber. The remaining cells on the upper surface of the membrane were removed with a cotton swab, and the cells on the lower surface of the membrane were migrating cells after 24 h of incubation at 37°C.The cells were stained with 0.1% crystal violet solution for 20 min after fixation with 4% paraformaldehyde for 30 min. The migrating cells were observed under a microscope and images were collected; five fields of migrating cells were randomly selected from each group for counting.

### Immunohistochemistry

GBM tissues were routinely embedded in paraffin and sectioned at a thickness of 6 μm. A xylene deparaffinization method was used for this process (33). The sections were treated with 10 mmol/L citrate buffer (pH 6.0) at 95°C for 20 min in a laboratory microwave for antigen retrieval and subsequently washed with 1× PBS. Endogenous peroxidase was blocked with 3% hydrogen peroxide solution, and the sections were blocked with goat serum (ZSGB-BIO, PV-9000). The sections were incubated with rabbit anti-human ADAMDEC1 polyclonal antibody (1:200, Abcam, USA) at 4°C on a shaker for 12 h, and washed three times with 1× PBS. The secondary antibody (1:100, Beyotime, USA) was added onto the sections at 37°C for 1 h. DAB staining, hematoxylin staining, microscope observation, and section analysis were used to evaluate the expression of ADAMDEC1. The study adhered to ethical standards and obtained the approval of an ethics committee.

### Statistical analysis

The mRNA expression data of gliomas and normal tissue samples were from The Cancer Genome Atlas (TCGA) database. All values are presented as the mean ± SEM. Significant differences were determined using GraphPad 5.0 software (USA). The Student’s *t*-test was used to determine significant differences between two groups. One-way ANOVA was used to determine statistical differences between multiple groups. The chi-square test was used to analyze the relationship between ADAMDEC1 expression and clinicopathological characteristics. Survival curves were plotted using the Kaplan–Meier method and compared by log-rank test. *p* < 0.05 was considered significant. All the experiments were repeated at least three times.

## Results

### Expression levels of the ADAM family in gliomas

**Figure 1:**
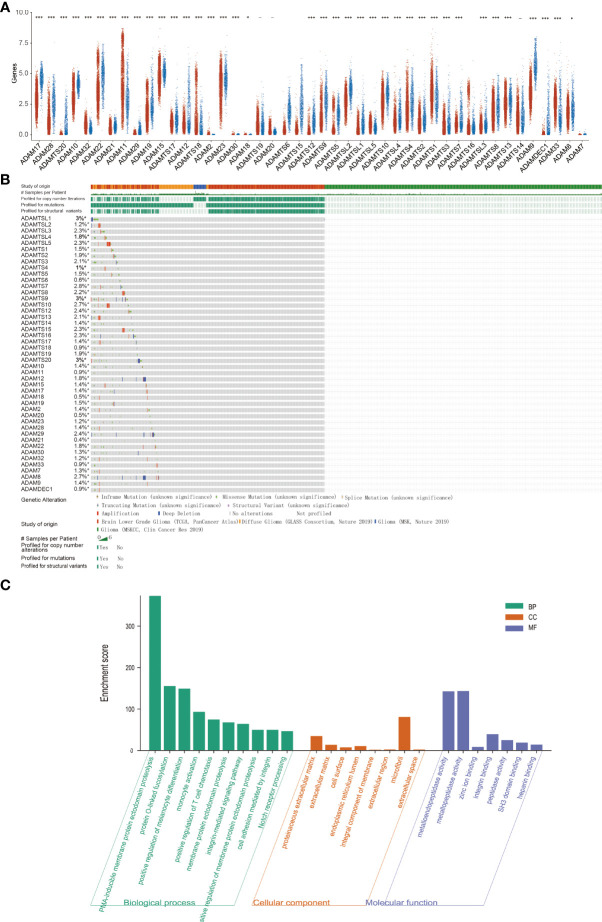
Expression analysis and functional analysis of ADAM family in gliomas. **(A)** The mRNA expression levels of ADAM family in gliomas. ADAMTS20 and ADAMDEC1 showed no expression in normal brain tissue, but their expression was significantly increased in gliomas. **(B)** Mutation probability of ADAM family in gliomas including inframe mutation, missense mutation, splice mutation, truncating mutation, and structural variant. **(C)** Biological processes, cellular component, and molecular function of ADAM family (GO analysis) showed that ADAM family affected the immune response and proliferation by regulating metalloendopeptidase activity, metallopeptidase activity, zinc ion binding, and so on. *p<0.05, ***p<0.001.

**Figure 2:**
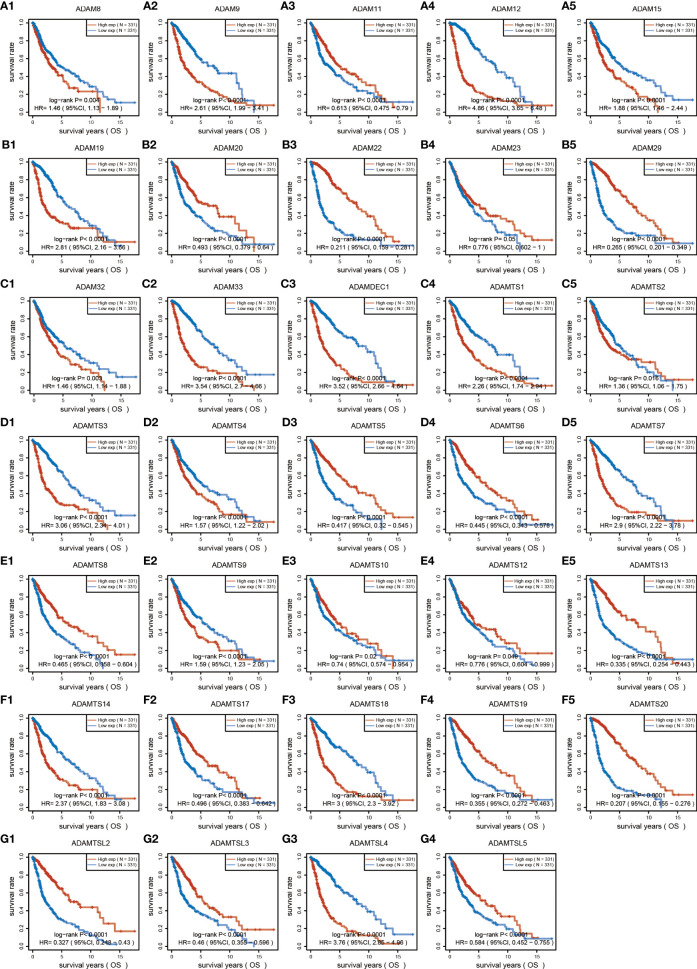
Overall survival analysis of ADAM family for gliomas. ADAM members (8, 9, 12, 15, 19, 32, 33), ADAMTS members (1, 2, 3, 4, 7, 9, 14, 18), ADAMTSL4, and ADAMDEC1 showed increased expression associated with poorer prognosis of GBM patients, while ADAM members (11, 20, 22, 23, 29), ADAMTS members (5, 6, 8, 10, 12, 13, 17, 19, 20), and ADAMTSL (2, 3, 5) showed decreased expression associated with poorer prognosis of glioma patients.

To investigate the role of the ADAM family of metalloproteinases in gliomas’ growth and progression, we analyzed the expression of the entire family for members in gliomas. We interrogated the bioinformatics database TCGA for mRNA expression levels of ADAM family members from 663 gliomas and 2,642 normal brains (**Figure 1A**). ADAM members (9, 10, 12, 17, 23, 28), ADAMTS (a disintegrin and metalloprotease with thrombospondin motifs) members (6, 7, 8, 9, 10, 12, 13, 15, 20), ADAMTSL1 (ADAMTS-like protein 1), and ADAMDEC1 were increased in GBM, while ADAM members (11, 15, 20, 32, 33) and ADAMTS members (1, 18) were decreased in GBM (**Figure 1A**). Meanwhile, ADAM members (2, 7, 18, 30) were merely expressed in normal brain and gliomas. Among them, ADAMTS20 and ADAMDEC1 showed no expression in normal brain tissue, but their expression was significantly increased in glioma (**Figure 1A**). The differences between the presence and absence of ADAMTS20 and ADAMDEC1 had obvious advantages in the prevention and identification of glioma in clinical treatment. Furthermore, the mutations of ADAM family members were assessed in gliomas, and ADAMTS20, ADAMTSL1 (ADAMTS-like protein 1), and ADAMTSL9 mutation probability was as high as 3%; other ADAM family members also had a series of mutations that were mainly concentrated in amplification (**Figure 1B**). GO analysis including biological process, cellular component, and molecular function was also shown in **Figure 1C**. The top 10 GO biological process terms suggested that ADAM family members were mainly involved in the inflammatory response like monocyte activation and positive regulation of T-cell chemotaxis, cell proliferation, and differentiation. Further investigation of these candidates using TCGA datasets revealed that ADAM members (8, 9, 12, 15, 19, 32, 33), ADAMTS members (1, 2, 3, 4, 7, 9, 14, 18), ADAMTSL4, and ADAMDEC1 showed increased expression associated with poorer prognosis of GBM patients, while ADAM members (11, 20, 22, 23, 29), ADAMTS members (5, 6, 8, 10, 12, 13, 17, 19, 20), and ADAMTSL (2, 3, 5) showed decreased expression associated with poorer prognosis of glioma patients (**Figure 2**). Among them, ADAM9, ADAM12, ADAMTS7, ADAMTS9, and ADAMDEC1 were the proteases where increased expression associated with poorer prognosis of glioma patients. TCGA data also demonstrated that the hazard ratio of overall survival was higher than 3.5 including ADAM12, ADAM33, ADAMTSL4, and ADAMDEC1 (**Figure S1**). ADAM12 and ADAMDEC1 have an obvious impact on the prognosis of glioma patients through comprehensive analysis including overall survival and ADAMs expression levels (**Figure 1A** and **Figure S1**). Combined with the presence and absence of ADAM family members’ expression in gliomas and the prognostic analysis, ADAMDEC1 had a great advantage in the prevention and treatment of gliomas, especially with the development of gene-targeted drugs.

### Identification and establishment of ADAMDEC1 prognostic signature in GBM

**Figure 3:**
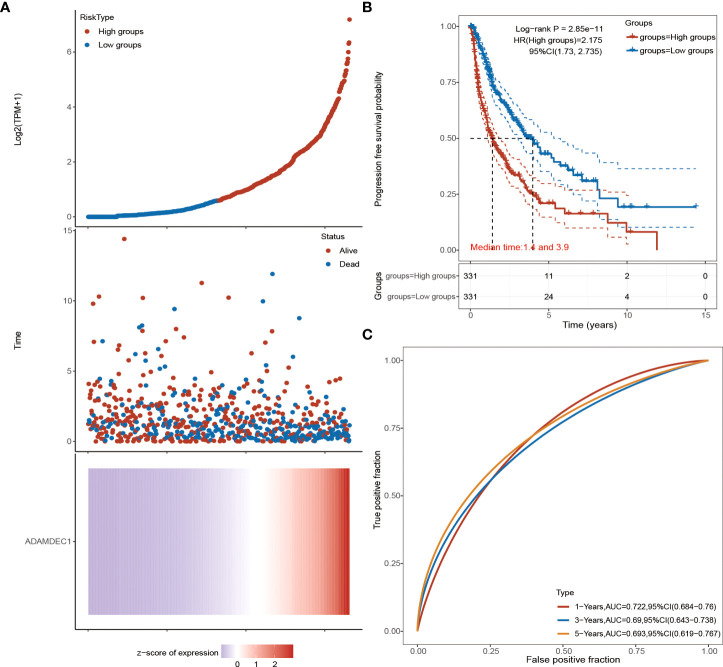
Advanced prognostic analysis of ADAMDEC1. **(A)** ADAMDEC1 prognostic analysis: the higher the expression of ADAMDEC1, the higher the risk score. **(B)** Progression-free survival probability analysis of ADAMDEC1 for gliomas. **(C)** Time-dependent ROC analysis of ADAMDEC1 signatures.

**Figure 4:**
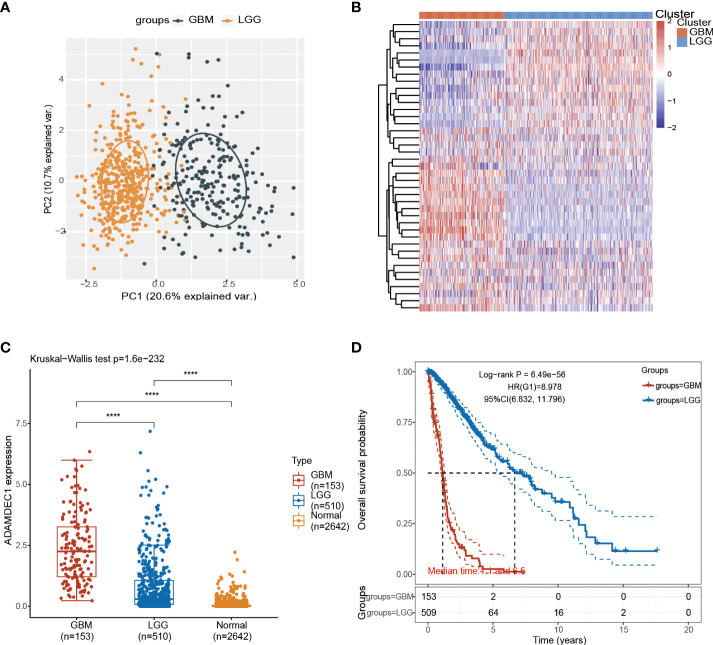
Analysis of glioma subtypes. **(A)** Glioma subtypes were divided into glioblastoma multiforme and low-grade gliomas of the brain. **(B)** Differential expression of ADAM family in GBM and LGG. **(C)** Differential analysis of ADAMDEC1 expression in GBM, LGG, and normal groups. **(D)** The higher the expression level of ADAMDEC1, the higher the tumor malignancy and the shorter the survival time. ****p<0.0001.

**Figure 5:**
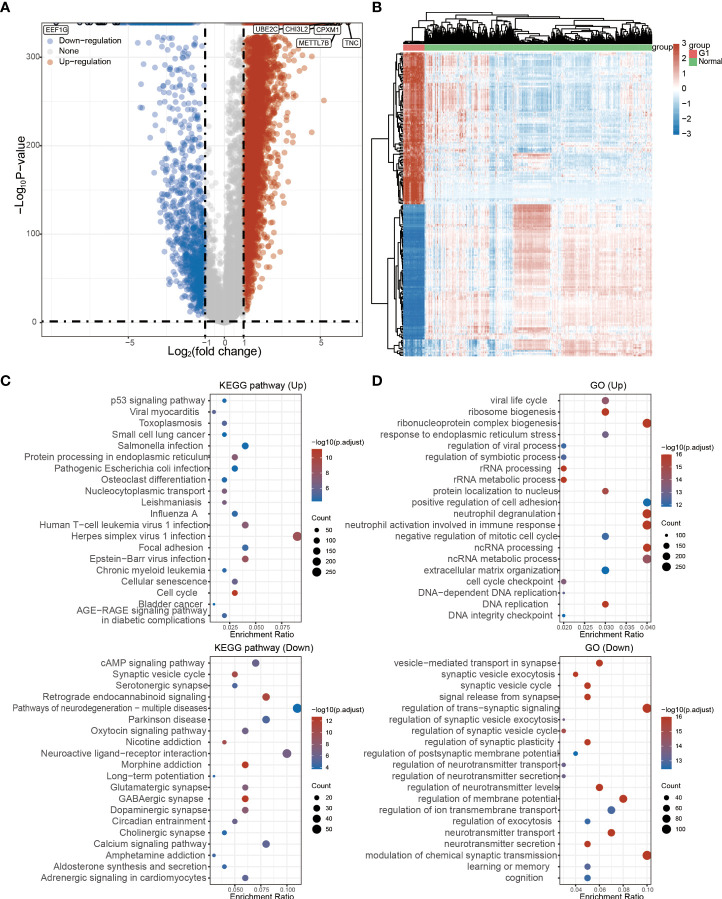
Functional analysis of ADAM family in glioma. **(A)** The regulated genes by the ADAM family in glioma. Each point represents a gene, red represents upregulated genes, blue represents downregulated genes, and gray represents genes that are not differentially expressed (FC > 2, *p* < 0.05). **(B)** Differentially expressed genes by the ADAM family in glioma. **(C)** KEGG pathway that was affected by the ADAM family in glioma. **(D)** GO terms of ADAM family function in glioma.

The detailed prognostic analysis of ADAMDEC1, which was related to poorer prognosis, is shown in **Figure 3A**. The dotted line represented the median risk score and divided the GBM patients into a low-risk and a high-risk group with the curve of risk score. Heatmap of the expression profiles of the prognostic genes in the low-risk and high-risk group. More dead glioma patients corresponding to the higher risk score had more obvious correlation to the expression of ADAMDEC1. Furthermore, progression-free survival (PFS) was also analyzed and the high ADAMDEC1 group represented poor PFS (**Figure 3B**). Time-dependent ROC analysis of the ADAMDEC1 signature is also shown in **Figure 3C**. The higher the AUC value, the stronger the predictive ability of the ADAMDEC1A. ADAMDEC1 showed stronger predictive ability in three stages of GBM. TCGA data also demonstrated ADAMDEC1 mRNA levels in different types of gliomas. Gliomas were divided into glioblastoma multiforme and brain lower-grade glioma according to the degree of malignancy (**Figure 4A**). The expression of ADAMDEC1 in the two subtypes was detected, and it was found that the expression of ADAMDEC1 was significantly different in GBM and LGG. Overall, the expression levels of most members of the ADAM family were higher in the GBM group than in the LGG group (**Figure 4B**). ADAMDEC1 showed a higher expression in the GBM group compared to the LGG group and the normal group. Meanwhile, ADAMDEC1 level was also higher in the LGG group than in the normal group. ADAMDEC1 expression level was higher in patients with GBM than in patients with LGG. We analyzed the expression of ADAMDEC1 in different subtypes and its effect on prognosis. We found the expression level of ADAMDEC1 to be as follows: GBM > LGG > normal; ADAMDEC1 showed that the higher the expression, the higher the malignancy of the tumor and the shorter the survival period (**Figures 4C, D**). Furthermore, the biological function of the ADAM family was analyzed in glioma, and we found that the ADAM family is differentially expressed in glioma (**Figures 5A, B**). The volcano plots to determine the expression profile between glioma and normal groups showed that there were 2,118 upregulated genes and 1,327 downregulated genes (**Figure 5A**). We then performed a hierarchical clustering of the top differentially expressed genes (FC > 2, *p* < 0.05) (**Figure 5B**). GO terms and KEGG analysis are shown in **Figures 5C, D**. KEGG analysis (the upregulated genes) showed that the ADAM family is rich in immune-related response, cell cycle, cell differentiation, and so on. The maximum saliency of the set ratio was the strongest (**Figure 5C**). The KEGG pathway controlled downregulated genes associated with the synthesis and delivery of various neurotransmitters, calcium signaling pathway, cAMP signaling pathway, and so on (**Figure 5C**). GO analysis of the differentially upregulated and downregulated expressed genes revealed that the ADAM family was enriched in the GO terms positive regulation of the ribonucleoprotein complex biogenesis, neutrophil degranulation, activation involved in immune response, regulation of trans-synaptic signaling, and modulation of chemical synaptic transmission, and there were strong significant differences (**Figure 5D**). In summary, the ADAM family mainly affected glioma *via* the immune response, cell cycle, neurotransmitters, and synaptic transmission.

### Immune cell infiltration of ADAMDEC1 in patients with GBM

**Figure 6:**
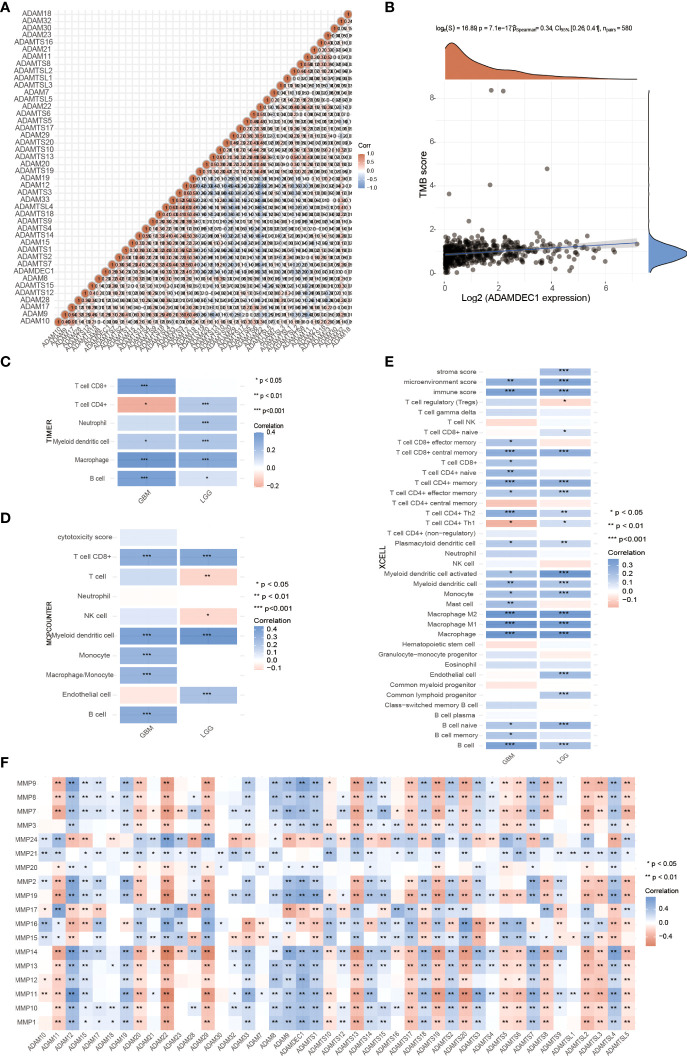
Effects of ADAMDEC1 on inflammation with GBM. **(A)** The correlation analysis of ADAM genes. **(B)** Correlation analysis between 622 patients with glioma expression and TM was performed using Spearman’s method. The abscissa represents gene expression distribution, and the ordinate represents TMB score distribution. The density curve on the right represents the distribution trend of TMB score; the upper density curve represents the distribution trend of ADAMDEC1 expression. The values on the top represent the correlation *p*-value (7.1e-17), correlation coefficient, and correlation calculation method. **(C–E)** Immune correlations: The heatmap of TIMER, MCPCOUNTER, and XCELL immune score and ADAMDEC1 expression in multiple tumor tissues (GBM and LGG). The abscissa represents different tumor tissues, and the ordinate represents different immune score. Different colors represent the correlation coefficients. Negative values indicate negative correlations and positive values indicate positive correlations; the deeper the color, the stronger the correlation. *p < 0.05, **p < 0.01, ***p < 0.001; asterisks (*) stand for significance levels. The statistical difference of two groups was compared through the Wilcox test. **(F)** Correlation between the expression of MMPs and ADAM family in gliomas.

These recent discoveries point to the ADAM family as a mediator of mechanisms underlying inflammation and as a possible therapeutic target for the prevention of inflammatory diseases. For example, ADAM-15 was found to be a mediator of rheumatoid arthritis and intestinal inflammation as well as inherent angiogenesis (6, 34). A disintegrin metalloprotease ADAM-like decysin-1 (ADAMDEC1) is an orphan ADAM-like metalloprotease that is believed to be closely related to inflammation. Firstly, we confirmed the correlation and interaction between ADAMDEC1 and other ADAM family members (**Figure 6A**). Tumor mutational burden (TMB) has received attention in immunotherapy, and ADAMDEC1 is an important biomarker for predicting response to antibody therapy (**Figure 6B**). Three immunoassays (TIMER, MCPCOUNTER, and XCELL) were used to determine whether ADAMDEC1 affects tumor progression through inflammatory responses in gliomas. we analyzed the role of ADAMDEC1 in inflammation (**Figures 6C–E**). ADAMDEC1 was positively correlated with B cells, CD8^+^ T cells, macrophages, and myeloid dendritic cells, and negatively correlated with CD4^+^ T cells. ADAMDDEC1 should present a different correlation in LGG that was positively correlated to B cells, CD4^+^ T cells, macrophages, and myeloid dendritic cells. The tumor microenvironment of GBM is infiltrated with various types of immune cells and cytokines. Macrophages are the most infiltrated immune cells in GBM, and most tumor-associated macrophages (TAMs) are considered to be immunosuppressive agents that are associated with tumor immune escape. Consistent with this, the ADAMDEC1 was highly positively related to the macrophage infiltration in the TME. Among the T cells, Tregs in the TME also play an immune-suppressive role to inhibit the T effector cells’ function. As shown in **Figure 6E**, the infiltration of Tregs was somewhat higher in the GBM, while it was negatively correlated with the expression of ADAMDEC1 in LGG. Moreover, the higher ADAMDEC1 expression also indicates more Th2 cells, another immune-suppressive CD4 subtype. However, higher ADAMDEC1 suggests more pro-inflammation Th1 cells in the TME in LGG, while less in the GBM. Taken together, the high expression of ADAMDEC1 helps to create an immune-suppressive TME and has an obvious correlation with disease stage.

**Figure 7:**
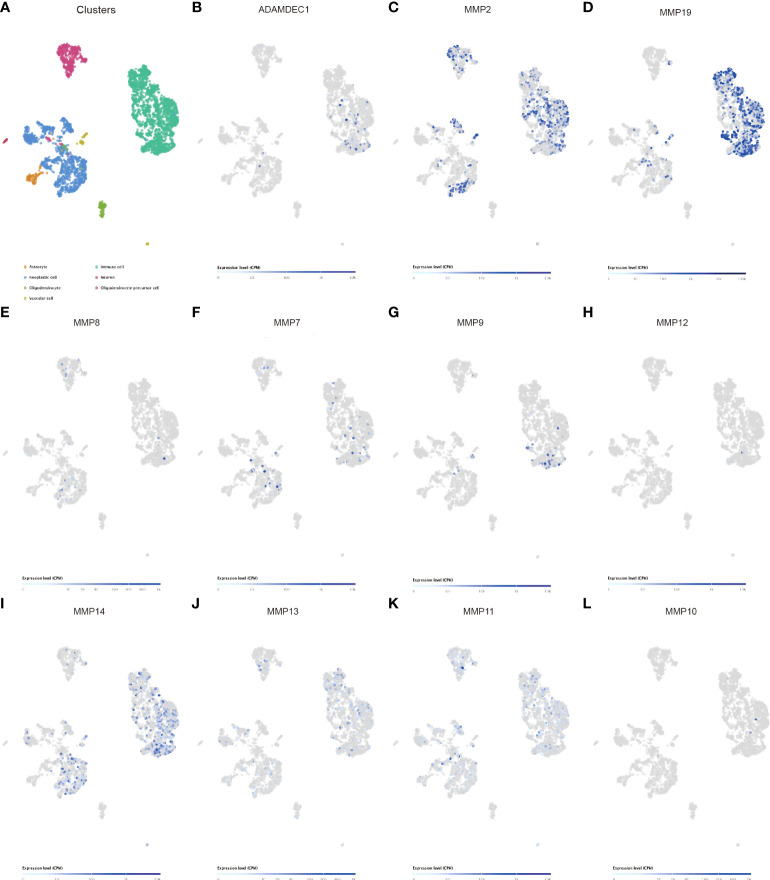
Single-cell sequencing analysis of ADAMDEC1 and other genes in human GBM. **(A)** Identification of the major cell populations in GBM. **(B–L)** Co-expression of ADAMDEC1 and MMPs in GBM.

Matrix metalloproteinases (MMPs) also play a major role in leukocyte infiltration and tissue inflammation. MMPs have been detected in cancer, and elevated MMP levels have been associated with tumor progression and invasiveness. MMPs are involved in initiation, proliferation, and metastasis of cancer through the breakdown of the extracellular matrix physical barriers. Overexpression of MMPs is associated with poor prognosis of cancer. Beyond that, studies have found that ADAM and MMPs jointly participate in numerous signaling pathways in our body, and play an important role in regulating neuroinflammation and tumors. For example, ADAM9 promotes the invasion and metastasis of cancer cells in melanoma by activating MMP1 and MMP2, while ADAM17 can activate MMP2 to treat the disease (prostate cancer and lung injury) by promoting angiogenesis (35, 36). ADAM17 targets MMP2 and MMP-9 *via* EGFR-MEK-ERK pathway activation to promote prostate cancer cell invasion. ADAM17/MMP inhibition prevents neutrophilia and lung injury in SARS-CoV-2-infected individuals to prevent the progression toward severe COVID-19 (37, 38). However, the role of ADAMDEC1 and MMP2 in cancer is still unclear. The correlation of MMPS and the ADAM family is shown in **Figure 6F**. We found that ADAMDEC1 was significantly positively correlated with MMP (2, 7, 8, 9, 10, 11, 12, 13, 14, and 19). To confirm the interaction between ADAMDEC1 and the MMPs, the single-cell sequencing analysis of gliomas showed that ADAMDEC1 had co-expression with MMP2 and MMP19 in immune cells (**Figure 7**). We speculate that ADAMDEC1 may co-regulate cell proliferation with MMP2 and MMP19.

### Silencing ADAMDEC1 abrogates the proliferation ability of glioma cells

**Figure 8:**
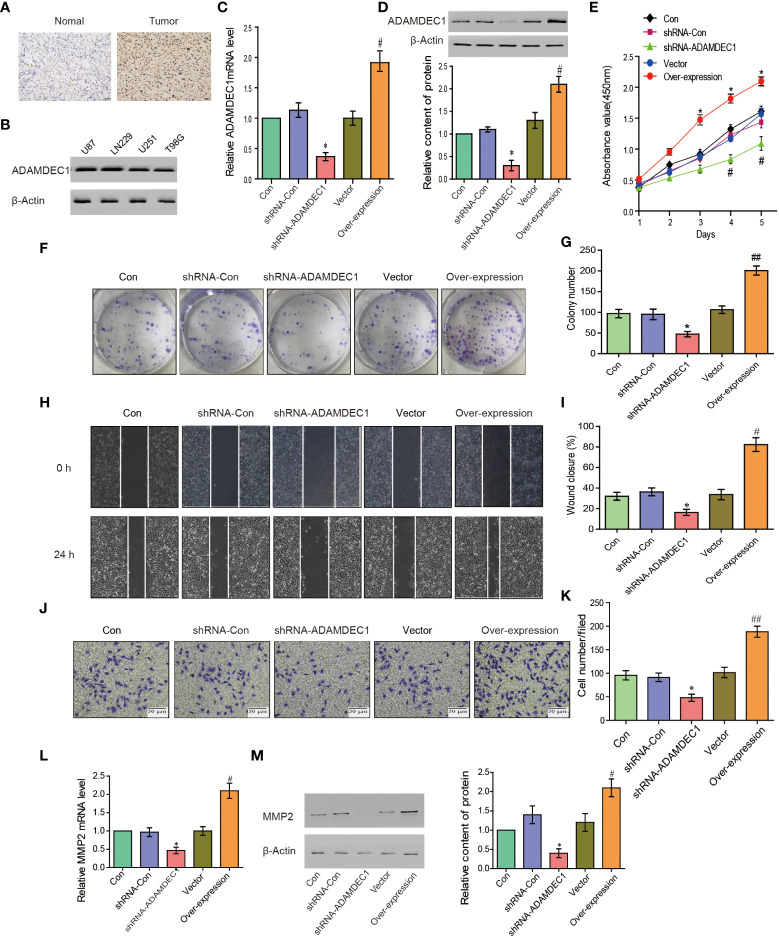
ADAMDEC1 promoted proliferation, migration, and invasion of GBM cells. **(A)** Immunohistochemical analysis of GBM samples showed that the signal of ADAMDEC1 in GBM was stronger than normal tissue (×400). **(B)** The levels of ADAMDEC1 expression in different cell lines of gliomas. **(C)** The mRNA levels of ADAMDEC1 in LN229 cells with different treatments (Con, shRNA-Con, shRNA-ADAMDEC1, vector, overexpression) in each group. **(D)** ADAMDEC1 protein levels in LN229 cells with different treatments (Con, shRNA-Con, shRNA-ADAMDEC1, vector, overexpression) in each group. **(E)** The proliferation of LN229 cells was detected by CCK-8, and overexpression of ADAMDEC1 promoted the proliferation of LN229 cells. **(F, G)** Colony formation experiments: Overexpression of ADAMDEC1 increased the average number of colonies. **(H, I)** Scratch test to detect the migration of LN229 cells (Con, shRNA-Con, shRNA-ADAMDEC1, vector, overexpression). **(J, K)** Invasion experiments to test the invasion of LN229 cells (Con, shRNA-Con, shRNA-ADAMDEC1, vector, overexpression) (×100). **(L)** Real-time PCR detection of MMP2 expression in LN229 cells (Con, shRNA-Con, shRNA-ADAMDEC1, vector, overexpression) in each group. **(M)** Western blot detection of MMP2 expression in LN229 cells (Con, shRNA-Con, shRNA-ADAMDEC1, vector, overexpression) in each group. Each bar represents the mean values ± SEM of three independent experiments. Compared to vector, ^#^ *p* < 0.05, ^##^ *p* < 0.01; compared to shRNA-Con. _*_p < 0.05.

The ADAM family and MMPS were widely expressed in various tumor cells and play a role in tumor genesis and invasion. To explore the biological roles of ADAMDEC1 in gliomas, immunohistochemical analysis of ADAMDEC1 expression in glioma tissues was further examined (**Figure 8A**). As shown in **Figure 8A**, ADAMDEC1 expression was primarily detected in the cytoplasm of glioma cells, and the staining intensity of ADAMDEC1 was increased in glioma tissues compared with normal tissues. Therefore, our findings indicated that the high expression of ADAMDEC1 was positively associated with advanced clinicopathological features in glioma patients. Then, we first examined ADAMDEC1 expression levels in glioma cell lines by Western blot, and LN229 of the glioma cell line was used in further experiments (**Figure 8B**). As shown in **Figures 8C, D**, the mRNA and protein levels of ADAMDEC1 were differentially decreased *via* knockdown ADAMDEC1 compared with shRNA-Con and increased *via* overexpression compared with vector.

To investigate the effects of overexpression or silencing of ADAMDEC1 on proliferation, migration, and invasion of glioma LN229 cells, we first used CCK-8 cell proliferation assay to confirm that overexpression of ADAMDEC1 enhanced cell proliferation and increased with time (*p* < 0.05). Knocking down ADAMDEC1 inhibited cell proliferation (**Figure 8E**). Colony formation assays revealed that silencing ADAMDEC1 dramatically inhibited the colony-forming ability of LN229 cells, while ADAMDEC1 overexpression promoted the growth of LN229 cells (**Figures 8F, G**). The effect of ADAMDEC1 on the migration of LN229 showed that overexpression of ADAMDEC1 significantly increased the mobility of LN229 cells, while silencing of ADAMDEC1 significantly decreased the mobility of LN229 cells (**Figures 8H, I**). Our results also showed that overexpression of ADAMDEC1 promoted the invasiveness of glioma LN229 cells, while the number of cells in the shRNA-ADAMDEC1 group was reduced (**Figures 8J, K**). These results showed that inhibiting ADAMDEC1 expression could effectively reverse the proliferation, migration, and invasion of glioma LN229 cells. As we found, ADAMDEC1 had co-expression with MMP2 in gliomas. Furthermore, we examined the effect of ADAMDEC1 on MMP2 expression levels. The mRNA and protein levels of MMP2 were differentially decreased *via* knockdown ADAMDEC1 compared with shRNA-Con; meanwhile, the mRNA and protein levels of MMP2 were significantly increased *via* overexpression of ADAMDEC1 compared with vector (**Figures 8L, M**). Taken together, the high expression of ADAMDEC1 correlated with advanced proliferation by regulating the MMP2 level.

## Discussion

The ADAM family has been shown to be involved in the occurrence of various diseases. ADAMDEC1, as a family member, has a unique active site and inhibits proteolytic activity because it is the only mammalian ADAM protease with a non-histidine zinc ligand (39). Glioblastoma is a very deadly disease with short survival times for patients, and due to the extremely aggressive and cellular resistance of tumor cells, complete surgical resection is impossible. Therefore, new targets are urgently needed to provide help for the prevention and treatment of glioma. ADAMDEC1 has been reported to participate in the maintenance of GBM cancer stem cells (GSCs), while targeted silencing of ADAMDEC1 reduced tumor cell proliferation (40). Liu et al. reported that knocking down ADAMDEC1 *in vitro* significantly inhibited the proliferation and invasion of glioma cells, and this inhibition effect might be through regulating apoptosis and cell-cycle-related proteins (41). Similarly, our study found that knockdown of ADAMDEC1 decreased the proliferation and colony-forming ability of LN229 cells, whereas ADAMDEC1 overexpression had opposite effects in LN229 cells *in vitro* (**Figures 8A–K**).

In our study, we first analyzed the expression of the ADAM family in glioma. Using the bioinformatics database TCGA, we found that ADAMDEC1 was not present in normal brain tissues compared with the tissues from glioma patients. The expression level was significantly increased in GBM, and the expression level in GBM was higher than LGG (**Figures 1A** and **4**). We further performed immunohistochemical analysis to investigate the effect of ADAMDEC1 on GBM and we found that ADAMDEC1 was highly expressed in GBM patients (**Figure 8A**). In addition, we also investigated the prognostic value of ADAMDECC1 in GBM. The results showed that the high expression of ADAMDEC1 was significantly associated with poorer OS (**Figure 2C3**, **Figure 3A**). Furthermore, we analyzed that the overall survival risk score of ADAMDEC1 was higher than the median risk score (**Figure S1**). In conclusion, ADAMDEC1 is a potential target for a diagnostic marker for GBM.

The ADAM family was considered as a mediator of mechanisms underlying inflammation. We assessed the correlation between ADAMDEC1 and cancer immune cell infiltration. ADAMDEC1 was positively correlated with B cells, CD8^+^ T cells, macrophages, and myeloid dendritic cells, and negatively correlated with CD4^+^ T cells. These results suggest that ADMDEC1 is involved in GBM progression by affecting immune cells (**Figure 6**). MMPs are proteins that play multiple roles in inflammation and are associated with tumor progression and invasion. The matrix metalloproteinase 2 (MMP2) gene is one of the members of the matrix metalloproteinase gene family (MMPs). TNF-α is released early in inflammation and IL-6 and other inflammatory mediators can promote the expression of MMP2, while overexpressed MMP2 can degrade the extracellular matrix, destroy the basal membrane, and further infiltrate inflammatory cells into deeper tissues. Tumor cells can also metastasize due to the destruction of the basal membrane and the dissolution of the extracellular matrix. These results suggest that MMP2 is closely related to inflammatory response (42, 43). Degradation of the extracellular matrix and basal membrane may further increase the infiltration of inflammatory cells, thereby exacerbating the inflammatory response.

Studies have found that MMP2 gene transcription and expression are closely related to the malignancy of various tumors, including GBM. Muniz-Bongers et al. found that MMP2 has a negative effect on the immune response in the tumor microenvironment. Knockdown of MMP2 can enhance the proliferation of T cells and the recruitment of NK cells, to inhibit the tumor growth (44). Sincevičiūtė et al. analyzed the formation and clinical results of MMP2 on gliomas at the RNA and protein levels, and concluded that MMP2 overexpression promoted the formation and invasion of gliomas and that the survival time of patients is shorter (45). In addition, MMP2 is associated with the malignancy and prognosis of breast cancer and lung cancer (46, 47). In addition, we also explored the molecular mechanism of ADAMDEC1 in tumor progression. Through single-cell sequencing, we found that ADAMDEC1 was co-expressed with MMP2 in immune cells (**Figure 7**). ADAMDEC1 was positively correlated with MMP2 (**Figures 8L, M**), and we predicted that ADAMDEC1 could regulate the proliferation and invasion of glioma cells by affecting the expression of MMP2.

Gliomas are aggressive, spread rapidly, and have a short survival time. In our study, it was shown that ADAMDEC1 is highly expressed in GBM, but hardly expressed in normal brain tissues. The high expression of ADAMDEC1 was significantly associated with advanced clinicopathological features and poor progression survival in glioma patients. In conclusion, this study provides evidence for ADAMDEC1 as a clinical biomarker and therapeutic target in GBM, and provides new insights into the development of immunotherapy drugs.

## Data availability statement

The datasets presented in this study can be found in online repositories. The names of the repository/repositories and accession number(s) can be found in the article/**Supplementary Material**.

## Ethics statement

The studies involving human participants were reviewed and approved by Ethics Committee of Weifang Medical University. The patients/participants provided their written informed consent to participate in this study.

## Author contributions

HQ, PW, and HS did experimental work. XL and JT constructed interfering lentivirus and overexpressing plasmid. XH, WT, LY, and JT performed analysis and analyzed results and data. JD and HW designed the research and wrote the manuscript. All authors contributed to the article and approved the submitted version.

## Funding

This work was financially supported by the National Natural Science Foundation of China (81602327 and 81500798), and the Zhishan Scholars Programs of Southeast University(2242021R41070).

## Conflict of interest

The authors declare that the research was conducted in the absence of any commercial or financial relationships that could be construed as a potential conflict of interest.

## Publisher’s note

All claims expressed in this article are solely those of the authors and do not necessarily represent those of their affiliated organizations, or those of the publisher, the editors and the reviewers. Any product that may be evaluated in this article, or claim that may be made by its manufacturer, is not guaranteed or endorsed by the publisher.

## Abbreviations

ADAM, a disintegrin and metalloprotease; ADAMDEC1, a disintegrin and metalloproteinase domain-like protein decysin-1; ADAMTS, a disintegrin and metalloprotease with thrombospondin motifs; ADAMTSL1 (ADAMTS-like protein 1), ADAMTS-like protein 1; GBM, glioblastoma multiforme; IDH, isocitrate dehydrogenase; LGG, lower-grade glioma; MMP2, matrix metalloproteinase 2; MMPs, matrix metalloproteinases; PFS, progression-free survival; TAMs, tumor-associated macrophages; TCGA, The Cancer Genome Atlas; TMB, tumor mutational burden; TME, tumor microenvironment; TNF-α, tumor necrosis factor; IL6, interleukin-6.

## References

[1] Louis DN Perry A Reifenberger G von Deimling A Figarella-Branger D Cavenee WK. The 2016 world health organization classification of tumors of the central nervous system: a summary. Acta Neuropathol (2016) 1316:803–20. doi: 10.1007/s00401-016-1545-1 27157931

[2] Jung E Alfonso J Osswald M Monyer H Wick W Winkler F. Emerging intersections between neuroscience and glioma biology. Nat Neurosci (2019) 2212:1951–60. doi: 10.1038/s41593-019-0540-y 31719671

[3] Bates EE Fridman WH Mueller CG. The ADAMDEC1 (decysin) gene structure: evolution by duplication in a metalloprotease gene cluster on chromosome 8p12. Immunogenetics (2002) 542:96–105. doi: 10.1007/s00251-002-0430-3 12037602

[4] Mullooly M McGowan PM Crown J Duffy MJ. The ADAMs family of proteases as targets for the treatment of cancer. Cancer Biol Ther (2016) 178:870–80. doi: 10.1080/15384047.2016.1177684 PMC5004698 27115328

[5] Kay J Thadhani E Samson L Engelward B. Inflammation-induced DNA damage, mutations and cancer. DNA Repair (Amst) (2019) 83:102673. doi: 10.1016/j.dnarep.2019.102673 31387777 PMC6801086

[6] Charrier-Hisamuddin L Laboisse CL Merlin D. ADAM-15: a metalloprotease that mediates inflammation. FASEB J (2008) 223:641–53. doi: 10.1096/fj.07-8876rev 17905725

[7] Paulissen G Rocks N Gueders MM Bedoret D Crahay C Quesada-Calvo F. ADAM-8, a metalloproteinase, drives acute allergen-induced airway inflammation. Eur J Immunol (2011) 412:380–91. doi: 10.1002/eji.200940286 21268008

[8] Cai Z Zhang A Choksi S Li W Li T Zhang XM. Activation of cell-surface proteases promotes necroptosis, inflammation and cell migration. Cell Res (2016) 268:886–900. doi: 10.1038/cr.2016.87 PMC4973336 27444869

[9] Mbikay M Mayne J Chretien M. The enigma of soluble LDLR: could inflammation be the key? Lipids Health Dis (2020) 191:17. doi: 10.1186/s12944-020-1199-9 PMC6998292 32014013

[10] Lambrecht BN Vanderkerken M Hammad H. The emerging role of ADAM metalloproteinases in immunity. Nat Rev Immunol (2018) 1812:745–58. doi: 10.1038/s41577-018-0068-5 30242265

[11] Jones JC Rustagi S Dempsey PJ. And gastrointestinal function. Annu Rev Physiol (2016) 78:243–76. doi: 10.1146/annurev-physiol-021014-071720 PMC4927194 26667078

[12] Scheller J Chalaris A Garbers C Rose-John S. ADAM17: a molecular switch to control inflammation and tissue regeneration. Trends Immunol (2011) 328:380–7. doi: 10.1016/j.it.2011.05.005 21752713

[13] Franzke CW Cobzaru C Triantafyllopoulou A Loffek S Horiuchi K Threadgill DW. Epidermal ADAM17 maintains the skin barrier by regulating EGFR ligand-dependent terminal keratinocyte differentiation. J Exp Med (2012) 2096:1105–19. doi: 10.1084/jem.20112258 PMC3371728 22565824

[14] Lisi S D'Amore M Sisto M. ADAM17 at the interface between inflammation and autoimmunity. Immunol Lett (2014) 1621 Pt A:159–69. doi: 10.1016/j.imlet.2014.08.008 25171914

[15] Murthy A Shao YW Narala SR Molyneux SD Zuniga-Pflucker JC Khokha R. Notch activation by the metalloproteinase ADAM17 regulates myeloproliferation and atopic barrier immunity by suppressing epithelial cytokine synthesis. Immunity (2012) 361:105–19. doi: 10.1016/j.immuni.2012.01.005 22284418

[16] Bulstrode H Jones LM Siney EJ Sampson JM Ludwig A Gray WP. A-disintegrin and metalloprotease (ADAM) 10 and 17 promote self-renewal of brain tumor sphere forming cells. Cancer Lett (2012) 3261:79–87. doi: 10.1016/j.canlet.2012.07.022 22841667

[17] Szalad A Katakowski M Zheng X Jiang F Chopp M. Transcription factor Sp1 induces ADAM17 and contributes to tumor cell invasiveness under hypoxia. J Exp Clin Cancer Res (2009) 281:129. doi: 10.1186/1756-9966-28-129 PMC2758847 19772640

[18] Łukaszewicz-Zając M Dulewicz M Mroczko B. A disintegrin and metalloproteinase (ADAM) family: Their significance in malignant tumors of the central nervous system (CNS). Int J Mol Sci (2021) 2219:10378. doi: 10.3390/ijms221910378 PMC8508774 34638718

[19] Sarkar S Zemp FJ Senger D Robbins SM Yong VW. ADAM-9 is a novel mediator of tenascin-c-stimulated invasiveness of brain tumor-initiating cells. Neuro Oncol (2015) 178:1095–105. doi: 10.1093/neuonc/nou362 PMC4490870 25646025

[20] Liu T Deng Z Xie H Chen M Xu S Peng Q. ADAMDEC1 promotes skin inflammation in rosacea *via* modulating the polarization of M1 macrophages. Biochem Biophys Res Commun (2020) 5211:64–71. doi: 10.1016/j.bbrc.2019.10.073 31627897

[21] Ha SE Jorgensen BG Wei L Jin B Kim MS Poudrier SM. Metalloendopeptidase ADAM-like decysin 1 (ADAMDEC1) in colonic subepithelial PDGFRα(+) cells is a new marker for inflammatory bowel disease. Int J Mol Sci (2022) 239 :5007. doi: 10.3390/ijms23095007 PMC9103908 35563399

[22] Zhang S Lv M Cheng Y Wang S Li C Qu X. Immune landscape of advanced gastric cancer tumor microenvironment identifies immunotherapeutic relevant gene signature. BMC Cancer (2021) 211:1324. doi: 10.1186/s12885-021-09065-z PMC8665569 34893046

[23] Hwang ES Lee HJ. Inhibitory effects of lycopene on the adhesion, invasion, and migration of SK-Hep1 human hepatoma cells. Exp Biol Med (Maywood NJ) (2006) 2313:322–7. doi: 10.1177/153537020623100313 16514180

[24] Macartney-Coxson DP Hood KA Shi HJ Ward T Wiles A O'Connor R. Metastatic susceptibility locus, an 8p hot-spot for tumour progression disrupted in colorectal liver metastases: 13 candidate genes examined at the DNA, mRNA and protein level. BMC Cancer (2008) 8:187. doi: 10.1186/1471-2407-8-187 18590575 PMC2488356

[25] Supiot S Gouraud W Campion L Jezéquel P Buecher B Charrier J. Early dynamic transcriptomic changes during preoperative radiotherapy in patients with rectal cancer: a feasibility study. World J Gastroenterol (2013) 1921:3249–54. doi: 10.3748/wjg.v19.i21.3249 PMC3671076 23745026

[26] Zhu W Shi L Gong Y Zhuo L Wang S Chen S. Upregulation of ADAMDEC1 correlates with tumor progression and predicts poor prognosis in non-small cell lung cancer (NSCLC) *via* the PI3K/AKT pathway. Thorac Cancer (2022) 137:1027–39. doi: 10.1111/1759-7714.14354 PMC8977174 35178875

[27] Tian W Wang P Wang Z Qi H Dong J Wang H. Phospholipid phosphatase 4 as a driver of malignant glioma and pancreatic adenocarcinoma. Front Oncol (2021) 11:790676:790676. doi: 10.3389/fonc.2021.790676 34917513 PMC8669803

[28] Chandrashekar DS Bashel B Balasubramanya SAH Creighton CJ Ponce-Rodriguez I Chakravarthi B. UALCAN: A portal for facilitating tumor subgroup gene expression and survival analyses. Neoplasia (New York NY) (2017) 198:649–58. doi: 10.1016/j.neo.2017.05.002 PMC5516091 28732212

[29] Goujon M McWilliam H Li W Valentin F Squizzato S Paern J. A new bioinformatics analysis tools framework at EMBL-EBI. Nucleic Acids Res (2010) 38(Web Server issue):W695–9. doi: 10.1093/nar/gkq313 PMC2896090 20439314

[30] Li T Fan J Wang B Traugh N Chen Q Liu JS. TIMER: A web server for comprehensive analysis of tumor-infiltrating immune cells. Cancer Res (2017) 7721:e108–e10. doi: 10.1158/0008-5472.Can-17-0307 PMC6042652 29092952

[31] Zhang X Li T Yang M Du Q Wang R Fu B. Acquired temozolomide resistance in MGMT(low) gliomas is associated with regulation of homologous recombination repair by ROCK2. Cell Death Dis (2022) 132:138. doi: 10.1038/s41419-022-04590-6 PMC8831658 35145081

[32] Yu X Liu W Wang Z Wang H Liu J Huang C. CD73 induces gemcitabine resistance in pancreatic ductal adenocarcinoma: A promising target with non-canonical mechanisms. Cancer Lett (2021) 519:289–303. doi: 10.1016/j.canlet.2021.07.024 34302921

[33] Paine MRL Ellis SR Maloney D Heeren RMA Verhaert P. Digestion-free analysis of peptides from 30-year-old formalin-fixed, paraffin-embedded tissue by mass spectrometry imaging. Anal Chem (2018) 9015:9272–80. doi: 10.1021/acs.analchem.8b01838 PMC6150647 29975508

[34] Sun C Beard RS Jr. McLean DL Rigor RR Konia T Wu MH. ADAM15 deficiency attenuates pulmonary hyperpermeability and acute lung injury in lipopolysaccharide-treated mice. Am J Physiol Lung Cell Mol Physiol (2013) 3043:L135–42. doi: 10.1152/ajplung.00133.2012 PMC3567368 23161886

[35] Oria VO Lopatta P Schilling O. The pleiotropic roles of ADAM9 in the biology of solid tumors. Cell Mol Life Sci (2018) 7513:2291–301. doi: 10.1007/s00018-018-2796-x PMC11105608 29550974

[36] Göoz P Göoz M Baldys A Hoffman S. ADAM-17 regulates endothelial cell morphology, proliferation, and *in vitro* angiogenesis. Biochem Biophys Res Commun (2009) 3801:33–8. doi: 10.1016/j.bbrc.2009.01.013 PMC2875258 19150341

[37] Xiao LJ Lin P Lin F Liu X Qin W Zou HF. ADAM17 targets MMP-2 and MMP-9 *via* EGFR-MEK-ERK pathway activation to promote prostate cancer cell invasion. Int J Oncol (2012) 405:1714–24. doi: 10.3892/ijo.2011.1320 22200661

[38] Lartey NL Valle-Reyes S Vargas-Robles H Jiménez-Camacho KE Guerrero-Fonseca IM Castellanos-Martínez R. ADAM17/MMP inhibition prevents neutrophilia and lung injury in a mouse model of COVID-19. J leukoc Biol (2021) 1116:1147–58. doi: 10.1002/jlb.3cova0421-195rr PMC9015574 34826347

[39] Lund J Olsen OH Sørensen ES Stennicke HR Petersen HH Overgaard MT. ADAMDEC1 is a metzincin metalloprotease with dampened proteolytic activity. J Biol Chem (2013) 28829:21367–75. doi: 10.1074/jbc.M113.474536 PMC3774404 23754285

[40] Jimenez-Pascual A Hale JS Kordowski A Pugh J Silver DJ Bayik D. ADAMDEC1 maintains a growth factor signaling loop in cancer stem cells. Cancer Discovery (2019) 911:1574–89. doi: 10.1158/2159-8290.Cd-18-1308 PMC7400732 31434712

[41] Liu X Huang H Li X Zheng X Zhou C Xue B. Knockdown of ADAMDEC1 inhibits the progression of glioma in vitro. Histol Histopathol (2020) 359:997–1005. doi: 10.14670/hh-18-227 32378728

[42] Andries L Masin L Salinas-Navarro M Zaunz S Claes M Bergmans S. MMP2 modulates inflammatory response during axonal regeneration in the murine visual system. Cells (2021) 107:1672. doi: 10.3390/cells10071672 PMC8307586 34359839

[43] Liang Y Yang N Pan G Jin B Wang S Ji W. Elevated IL-33 promotes expression of MMP2 and MMP9 *via* activating STAT3 in alveolar macrophages during LPS-induced acute lung injury. Cell Mol Biol Lett (2018) 23:52. doi: 10.1186/s11658-018-0117-x 30410547 PMC6208075

[44] Muniz-Bongers LR McClain CB Saxena M Bongers G Merad M Bhardwaj N. MMP2 and TLRs modulate immune responses in the tumor microenvironment. JCI Insight (2021) 612:e144913. doi: 10.1172/jci.insight.144913 PMC8262464 34032639

[45] Sincevičiūtė R Vaitkienė P Urbanavičiūtė R Steponaitis G Tamašauskas A Skiriutė D. MMP2 is associated with glioma malignancy and patient outcome. Int J Clin Exp Pathol (2018) 116:3010–8. PMC6958083 31938426

[46] Han L Sheng B Zeng Q Yao W Jiang Q. Correlation between MMP2 expression in lung cancer tissues and clinical parameters: a retrospective clinical analysis. BMC Pulm Med (2020) 201:283. doi: 10.1186/s12890-020-01317-1 PMC7594265 33115469

[47] Jiang H Li H. Prognostic values of tumoral MMP2 and MMP9 overexpression in breast cancer: a systematic review and meta-analysis. BMC Cancer (2021) 211:149. doi: 10.1186/s12885-021-07860-2 PMC7877076 33568081

