# Supplementary figures and images for: ADAMDEC1 accelerates GBM progression via activation of the MMP2-related pathway
Source: Front Oncol. 2022 Sep 12;12:945025. doi: 10.3389/fonc.2022.945025 (PMC9511150; doi:10.3389/fonc.2022.945025)

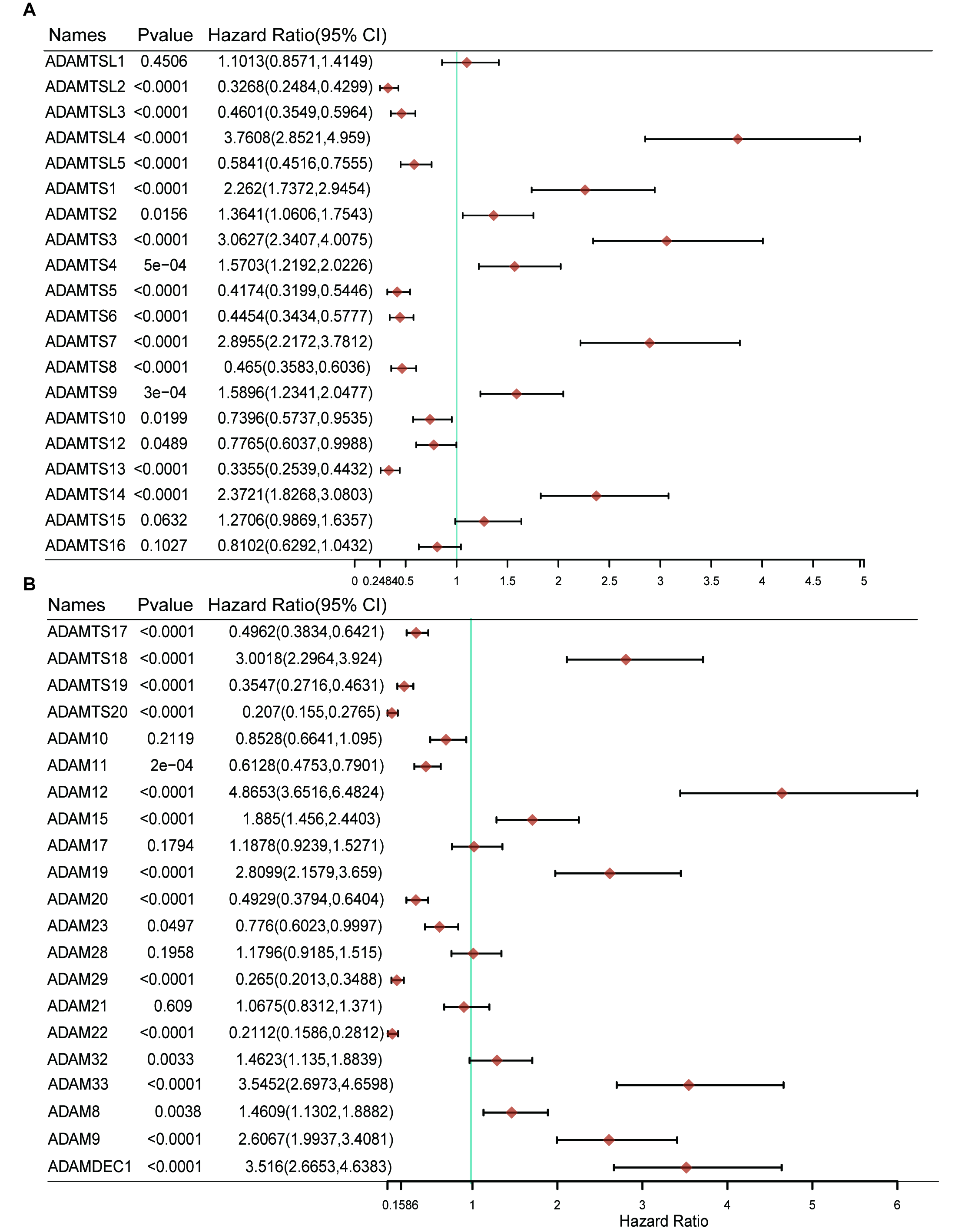

Supplement: Supplementary file 1 [file Image_1.tif]

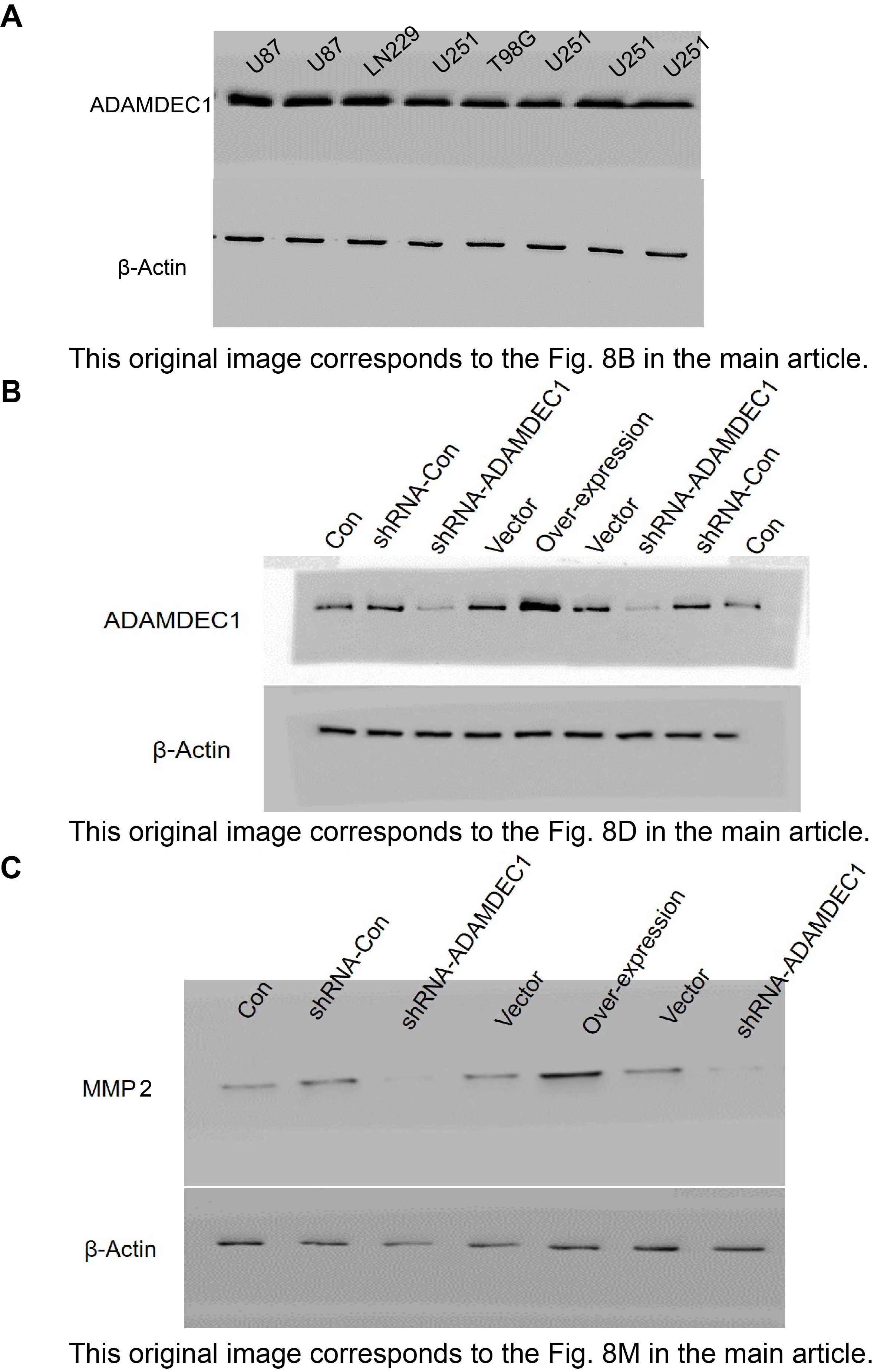

Supplement: Supplementary file 2 [file Image_2.jpg]
